# Flame Spray Pyrolysis Co_3_O_4_/CoO as Highly-Efficient Nanocatalyst for Oxygen Reduction Reaction

**DOI:** 10.3390/nano11040925

**Published:** 2021-04-05

**Authors:** Loukas Belles, Constantinos Moularas, Szymon Smykała, Yiannis Deligiannakis

**Affiliations:** 1 Laboratory of Physics Chemistry of Materials & Environment, Department of Physics, University of Ioannina, 45550 Ioannina, Greece; loukasbelles@gmail.com (L.B.); k.moularas@uoi.gr (C.M.); 2 Institute of Engineering Materials and Biomaterials, Silesian University of Technology, 18a Konarskiego St, 44-100 Gliwice, Poland; szymon.smykala@polsl.pl

**Keywords:** Oxygen Reduction Reaction (ORR), Flame Spray Pyrolysis (FSP), cobalt oxide, nanomaterials, fuel cells, rotating disc electrode (RDE)

## Abstract

The oxygen reduction reaction (ORR) is the rate-limiting reaction in the cathode side of fuel cells. In the quest for alternatives to Pt-electrodes as cathodes in ORR, appropriate transition metal oxide-based electrocatalysts are needed. In the present work, we have synthesized Co_3_O_4_ and CoO/Co_3_O_4_ nanostructures using flame spray pyrolysis (FSP), as electrocatalysts for ORR in acidic and alkaline media. A detailed study of the effect of (Co-oxide)/Pt ratio on ORR efficiency shows that the present FSP-made Co-oxides are able to perform ORR at very low-Pt loading, 0.4% of total metal content. In acid medium, an electrode with (5.2% Pt + 4.8% Co_3_O_4_), achieved the highest ORR performance (J_max_ = 8.31 mA/cm^2^, E_1/2_ = 0.66 V). In alkaline medium, superior performance and stability have been achieved by an electrode with (0.4%Pt + 9.6% (CoO/Co_3_O_4_)) with ORR activity (J_max_ = 3.5 mA/cm^2^, E_1/2_ = 0.08 V). Using XRD, XPS, Raman and TEM data, we discuss the structural and electronic aspects of the FSP-made Co-oxide catalysts in relation to the ORR performance. Cyclic voltammetry data indicate that the ORR process involves active sites associated with Co^3+^ cations at the cobalt oxide surface. Technology-wise, the present work demonstrates that the developed FSP-protocols, constitutes a novel scalable process for production of co-oxides appropriate for oxygen reduction reaction electrodes.

## 1. Introduction

Flame spray pyrolysis (FSP) is an established versatile one-step process for scalable synthesis of metal-oxide nanocrystals, with precisely controlled properties [1]. During the last two decades, FSP technology has been explored for synthesis of various types of nanomaterials e.g., metal-oxides, metallic particles, and heterojunctions [2,3] to name a few. Control of the combustion-flame characteristics, i.e., reactor configuration, reactants composition, temperature profiles, allow control of particle composition [4,5], size [4,5], morphology [5,6], defects [3]. These factors are known to be crucial for optimal catalytic or electrocatalytic performance [7,8].

In fuel cells cathodes, the one-electron reduction of O_2_ to form OO^¯^ (ORR) is thermodynamically non-favorable [9]. As a result, the ORR process is not spontaneous, therefore an appropriate catalyst is mandatory, which usually requires a non-zero voltage bias [7,10]. Usually, ORR can proceed via two well-known pathways, i.e., either two-, or four-electrons pathway [11]. The preferable path strongly depends on factors such as pH of the electrolyte [12], operation temperature [13], and mostly on the type of catalytic material [4,7,11,14]. Equation (1a–f) summarize the pertinent electron transfer reactions [15,16] for Pt^0^ which is the benchmark material in ORR research [17,18], at acidic or alkaline pH respectively [9,12].

### 1.1. Alkaline Medium

Direct Pathway (4 e^−^ process)

(1a) $$O_{2}+2H_{2}O+4e^{-}\to 4{\mathrm{OH}}^{-}{\;E}^{0}=0.401\;V$$

Sequential Pathway-Step a (2 e^−^ process):

(1b) $$O_{2}+2H_{2}O+2e^{-}\to {OOH}^{-}+{\mathrm{OH}}^{-}{\;E}^{0}=-0.065\;V$$

Sequential Pathway-Step b (2 e^−^ process):

(1c) $${\mathrm{OOH}}^{-}+H_{2}O+2e^{-}\to 3{\mathrm{OH}}^{-}{\;E}^{0}=0.867\;V$$

### 1.2. Acidic Medium

Direct Pathway (4 e^−^ process)

(1d) $$O_{2}+4H^{+}+4e^{-}\to 2H_{2}{O\;E}^{0}=1.229\;V$$

Sequential Pathway-Step a (2 e^−^ process)

(1e) $$O_{2}+2H^{+}+2e^{-}\to H_{2}O_{2}{\;E}^{0}=0.695\;V$$

Sequential Pathway-Step b (2 e^−^ process)

(1f) $$H_{2}O_{2}+2H^{+}+2e^{-}\to 2H_{2}{O\;E}^{0}=1.763\;V$$

When the overall efficiency of a fuel-cell is considered, the two-electron pathway usually results to lower efficiency than the four-electron, therefore typically the 4 e^−^ pathway is desired to achieve high-efficiency in fuel cells [9]. As described in Equation (1), the electron-transfer reaction pathways can be either direct or sequential [19], i.e., the sequential pathway is not a necessary intermediate for the direct pathway. At acidic-medium in the sequential-reaction scheme, the formation of hydrogen peroxide, H_2_O_2_, intervenes Equation (1e,f). This is non-desirable, thus the sequential-process in acid-medium should be suppressed [8,19]. In alkaline medium, the key-intermediate is an OOH^−^ anion, Equation (1b,c). The rate-limiting steps in ORR can be determined by [a] the adsorption-events of O_2_ or OOH_(ads)_ [20] in acid or alkaline medium, or [b] the desorption of the formed hydroxyl-anions OH^−^, that can be in the form of H_2_O (i.e., H^+^ + OH at acidic medium) or OH^−^ (alkaline medium) [21]. According to He and Cairns [9], the overpotential required for ORR is correlated with the difference of equilibrium potential between the {first electron transfer} and {overall reaction}. Thus, eligible catalysts for ORR should cope with all these rate-limiting factors, in addition they should poses appropriate stability.

Today, state-of-the-art ORR cathodes are made by noble metals like platinum [11], palladium [22], their alloys [23], or bimetallic catalysts [24]. Typically, the best-performing catalytic noble metals are nanosized, usually supported on an appropriate conductive carbon-support [25,26]. So far, platinum/carbon (Pt^0^/C) cathodes are the most promising candidates for ORR, in both alkaline and acidic media [27], due to their good electrocatalytic activity [28] and stability [29]. However, the high-cost and environmental scarcity of Pt are prohibitive factors, thus, low-cost/earth-abundant metal oxide catalysts are at immediate need. Moreover, the properties of the carbon-support, i.e., such as graphitization quality, conductivity, O_2_ diffusion and surface area affect the ORR activity and catalyst’s durability. So far, only few eligible non-noble metals have been reported, i.e., most reported cases display inferior ORR performance vs. Pt [30,31]. An alternative strategy is to develop alloys of Pt with inexpensive metals e.g., Ni, Co, Cu, or Fe [30]. The premise of this approach is that, if efficient ORR can be achieved, this could be combined with improvement of the stability of the Pt cathode, since Pt suffers from dissolution or agglomeration [15]. In this direction, {noble/non-noble metal} alloys have been extensively studied as ORR cathodes in acidic media [31].

**Figure 1:**
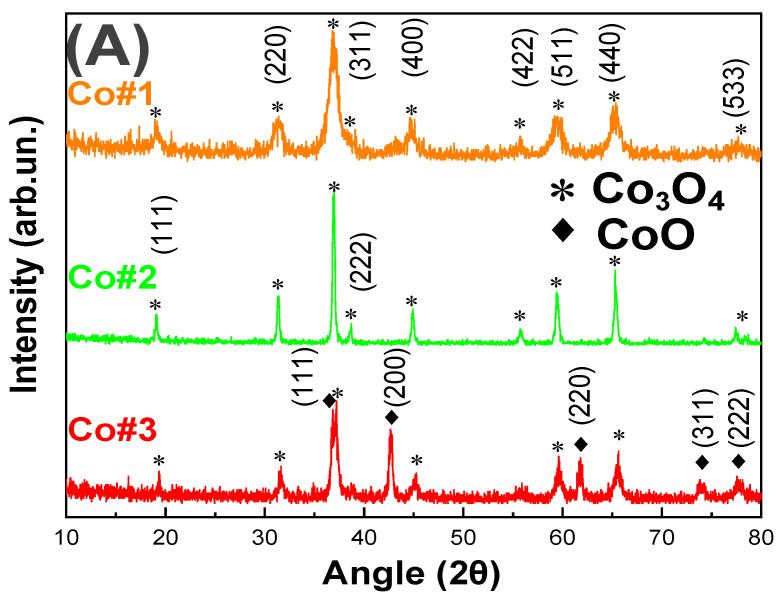

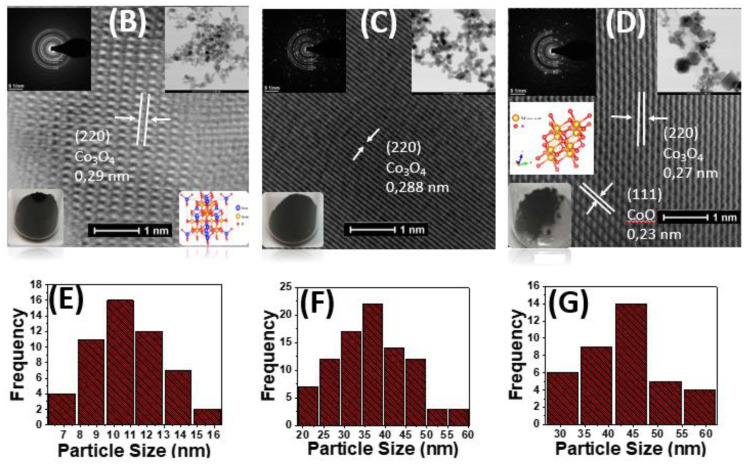
TEM images and (**A**) XRD pattern of our #Co1, #Co2 and #Co3 nanomaterials. (**B**) As prepared Co_3_O_4_, #Co1, (**C**) Calcined Co_3_O_4_, #Co2 and (**D**) CoO@Co_3_O_4_ particles #Co3. Insets: SAED pattern of #Co1, #Co2 #Co3 respectively and photos of the powders. Size pattern distribution histograms of (**E**) #Co1 (**F**) #Co2 (**G**) #Co3.

Recently, encouraging results on non-noble metal-oxides have been reported for ORR cathodes [32]. Theoretical and experimental studies of some transition metal oxides (TMOs) [19], i.e., cobalt oxides [33], copper oxides [34], manganese oxides [35] and iron oxides [36], Some mixed transition metal oxides, i.e., ZnCo_2_O_4_/C [37], CoCuMnO_x_ [38], CoO/MnO_2_ [39] show encouraging results on ORR efficiency [40], while a study on Co_x_Si_y_O_z_ reported a highly satisfactory stability [41]. Among TMOs, cobalt-oxide is a promising candidate, thanks to its low price, with ability to be stabilize Co^3+^/Co^2+^ oxidation-states [42]. More particularly Co_3_O_4_ is a spinel-type {2Co^3+^-1Co^2+^} structure, where Co^3+^-cations occupy octahedral sites and Co^2+^-cations occupy tetrahedral sites [43], see inset in Figure 1B. In principle, the occurrence of the Co^2+^/Co^3+^ redox couple in the Co_3_O_4_ unit-cell, provides an option for electron transfer [43]. In this context, cobalt-oxide nanoparticles supported on graphene [30] or carbon nanotubes [44] have been reported as efficient ORR cathode electrodes. However, their performance was satisfactory only in alkaline medium, not in acidic medium [19,45]. The issue of efficient ORR operation at both acidic and alkaline medium remains a cumbersome challenge [19,46] that requires diligent control of interfacial adsorption-desorption of O-species/electrons and protons [46].

In this context, specific aims of the present work were: (1) using FSP-technology to synthesize Co-oxide nanomaterials optimized for ORR at both acidic and alkaline environment, (2) to construct efficient ORR electrodes with very-low Pt-content, operating at both alkaline and acid medium, and (3) to investigate the role of nanoparticle properties, with specific emphasis on phase composition (Co_3_O_4_/CoO) and Co^2+^/Co^3+^ redox speciation.

## 2. Materials and Methods

Chloroplatinic acid hexahydrate (H_2_PtCl_6_·6H_2_O, Sigma-Aldrich, (Saint Louis, MO, USA), Sodium Borohydride (NaBH_4_), used to form the Pt nanoparticles were purchased from Sigma Aldrich (Saint Louis, MO, USA). Carbon black (Vulcan XC-72R) was a kind-gift from Cabot (Boston, MA, USA), was used as-received as the support of the electrocatalysts.

### 2.1. Synthesis of Co-Based Nanocatalysts by Flame Spray Pyrolysis

Our lab-scale FSP reactor, used to produce the Co-oxides, has been described in detail previously [6,46]. To prepare Co_3_O_4_, a 0.3 M Co-precursor solution was prepared by dissolving cobalt acetate in a mixture of solvents consisting of 50% acetic acid, 40% iso-octane, 10% 2-butanol. In FSP-process, the high combustion enthalpy of iso-octane (5460 kJ/mol) increases the high temperature regime and flame length, thus promoting the desired gas-to-particle formation [3]. The precursor fuel (P) was supplied to the nozzle through a syringe pump at a feed rate of 5 mL/min and atomized into fine droplets, using a dispersion oxygen (D) flow of 5 L/min, i.e., P/D = 5/5, at a pressure-drop of 1.5 bar. The spray-ignition was initiated and sustained by a surrounding, supporting flamelet ring of premixed O_2_/CH_4_ (3/1.5 L/min). The CoO/Co_3_O_4_ heterostructure was produced by increasing the fuel in the flame to P = 8 mL/min. This {precursor to dispersion} ratio P/D = 8/5, resulted in a slightly O-lean flame, i.e., φ = 0.9 thus promoting the suboxic CoO phase [47]. The flame flow-directionality was assisted by an additional 5 L/min sheath O_2_, provided thorough a toroidal-ring with 16 holes, 500 μm diameter each, located annularly at 2 cm around the nozzle tip. A vacuum pump (Busch V40) was used to collect the particles on a glass microfiber filter (Albeit), at a Burner-to-Filter Distance BFD = 60 cm, above the flame. The product powder was collected from the filter by scraping. The so prepared Co-oxide materials are herein codenamed as #Co1, #Co2, and #Co3 for the as-prepared Co_3_O_4_, the calcined (500 °C, 8 h), and the Co_3_O_4_/CoO material, repressively, see Table 1.

### 2.2. Characterization of Nanocatalysts

X-Ray Diffraction (XRD): The crystal structures of the nanocatalysts were analyzed by XRD in a Bruker Advance D8 diffractometer (Cu Ka radiation λ = 1. 5406 Å, 40 kV, 40 mA) at 2θ = 10°–80° (step size of 0.03° at a rate of 2 s per step). The average crystallite size of FSP-made particles was calculated by the Scherrer equation [48]

(2) $$d_{XRD=}\frac{k\lambda }{\beta \left(\cos\theta \right)}$$

where *d_XRD_* is the crystallite size (nm), *k* is a shape constant (in this case 0.9), *λ* is the wavelength of Cu Kα radiation (1.5406 Å), *β* is the full width at half maximum and *θ* is the peak-diffraction angle.

Brunauer–Emmett–Teller (BET) Analysis: The specific-surface-area (SSA, m^2^/gr) of the synthesized materials was determined by the N_2_ adsorption-desorption method [49] at 77 K using a Quantachrome Autosorb-1 instrument (Bounton Beach, FL, USA). To acquire the BET isotherms, powders were degassed for 2 h at 120 °C, in flowing N_2_ over a relative pressure range of P/P_0_ = 0−1. An average primary particle size, d*_BET_*, was calculated by the estimated SSA assuming monodisperse spheres according to the Equation (3) [50]

(3) $$d_{BET}=\frac{6}{\rho_{p}\cdot SSA}$$

where *ρ_p_* is the weighted density of the particles with *ρ* [Co_3_O_4_] = 5.18 g/cm^3^ and *ρ* [CoO] = 6.44 g/cm^3^ [51].

X-Ray Fluorescence (XRF): Sample excitation was performed with an annular 109 Cd radio-isotopic source (RITVERC GmbH). X-ray source had a radius of 12.5 mm housed in a cylindrical container, fixed coaxially above a CANBERRA SL80175 Si(Li) detector (5 mm crystal thickness, 80 mm^2^ area), with a 25 μm-thick Be window and an energy resolution f 171 eV for the 5.9 keV Mn Kα line. Data acquisition was performed using a PCI card, controlled by the ORTEC MAESTRO−32 software, and spectral analysis was carried out using the WinQxas software package (International Atomic Energy Agency La-boratories Seibersdorf, XRF Group, Seibersdorf (Austria), IAEA 1997–2002).

Raman Spectroscopy: Raman spectroscopy measurements were performed with a HORIBA XploRA PLUS instrument (Kyoto, Japan) with a 785 nm diode laser as excitation source, focused with a microscope. The materials were pressed into pellets and placed on a glass plate. Each Raman spectrum was recorded performing 30 accumulations in 10 s, to obtain adequate signal-to-noise ratio.

TEM: The morphology of the materials was analyzed by transmission electron microscopy using a FEI Titan 80–300 S/TEM microscope at 300 kV accelerating voltage and a 21.5 mrad beam convergence angle. Before the measurements, the nanopowders were dispersed in ethanol, sonicated at a bath sonicator and then deposited as single droplet of suspension on a copper TEM grid covered with a thin carbon layer. Selected area electron diffraction patterns were acquired with the same instrument in TEM mode, under parallel electron beam illumination.

X-Ray Photoelectron Spectroscopy (XPS) data were acquired in a surface analysis ultrahigh vacuum system (SPECS GmbH) equipped with a twin Al-Mg anode X-ray source and a multichannel hemispherical sector electron analyzer (HSA-Phoibos 100). The base pressure was 2–5 × 10^−9^ mbar. A monochromatized Mg Kα line at 1253.6 eV and an analyzer pass-energy of 15 eV were used in all measurements. The binding energies were calculated with reference to the energy of C1 s peak of adventitious carbon at 284.5 eV. The peak deconvolution was calculated using CasaXPS software with a Shirley background.

### 2.3. Preparation of Electrocatalytic Working Electrodes

Pt^0^-particles were synthesized as a reference catalyst, employing the wet-impregnation method [52], based on formation of fine Pt^0^ particles via reduction of Pt^2+^ by BH_4_. Briefly, 85 mg of the Pt-salt was dissolved in 8 mL of ultra-pure triple distilled water (Millipore SIMS600 CP Burlington, USA) at room temperature, T = 23 °C, plus 320 mg of Vulcan XC-72 R carbon black. The mixture was allowed to mix under continuous stirring for 2 h, until it became a homogeneous black slurry. Then, NaBH_4_ was added dropwise to the Pt/carbon slurry, using a 10 μL pipet, at a rate of 1 mL/min under vigorous stirring. The NaBH_4_ was taken from a stock of 31 mg NaBH_4_ dissolved in 8 mL of triple distilled water. The reduction potential of the NaBH_4_/Pt/carbon slurry was E_h_ = −65 mV vs. NHE, Ref. [53] monitored in-situ by a redox Pt-electrode (Metrohm, Pt Working Electrode 3 mm diameter Herisau, Switzerland). Then, the resulting NaBH_4_ /Pt/carbon slurry was heated under stirring for 12 h at 90 °C, and afterwards the formed solid residue was collected by centrifugation at 6000 rpm. The collected solid was dried for 5 h at 23 °C under a N_2_ stream. This material, herein codenamed as {10 wt% Pt/C}, had a Pt-loading of 10.1% as determined by XRF.

The electrocatalytic materials were deposited as drop-casted films, on a glassy-carbon disk electrode (working electrode RDE.GC30 by Metrohm Autolab B.V.), with a 3 mm diameter (with a geometric surface area of 0.071 cm^2^), mounted on a RDE holder (RDE−2 Metrohm Autolab B.V.). To construct the electrode, first a catalyst-suspension was prepared containing 7 mg of the material-powder in 6 mL mixture of triple distilled water (2.75 mL) and isopropanol (3.25 mL) (Merck, ACS Reag, New Jersey, USA) [29]. Depending on the experiment, the catalyst-powder was either Pt^0^-particles, or FSP-made Co-oxide particles, or appropriate mixtures of {Pt^0^-particles/plus FSP-made Co-oxide particles}.

Before the drop-casting step, the catalyst mixture was ultrasonicated using a 20 W ultrasonication bath (Elmasonic S10 h, Singen, Germany) to achieve a homogeneous slurry [54]. Deposition of each electrocatalyst material on the working disk-electrode was performed by drop-casting of the prepared suspension onto the glassy-carbon disk. Before drop-casting each glassy electrode was polished with aluminium oxide powder (grain size 0.3 μm) on a polishing cloth, as recommended by Metrohm. Then, 25 μL of the catalyst suspension was drop-casted on to the glassy carbon, using a micro-pipette (1 μL per drop), and rapidly dried at room temperature 23 °C, forming a dry {catalyst/carbon} film on the glassy-carbon. Then, 3 μL of Nafion-solution was drop-casted on the formed film and allowed to dry at room temperature 23 °C, forming a dry {Nafion:catalyst/carbon} film on the glassy-carbon. The Nafion-solution consisted of 5 wt% perfluorinated Nafion resin solution (Sigma Aldrich, MO, USA), in triple distilled water/isopropanol (110 μL Nafion solution: 5.5 mL triple distilled water: 6.5 mL isopropanol). According to the standard procedure, the deposition of a Nafion on top of the catalyst film acts as a binding agent [55] stabilizing the electrode.

### 2.4. Catalytic ORR Evaluation

All ORR experiments were carried out at room temperature, 23 °C, in a three-compartment-cell, filled with 50 mL triple distilled water containing either 0.1 M H_2_SO_4_ for acidic media measurements or 0.1 M NaOH for alkaline media measurements. An Ag/AgCl (3 M KCl) electrode (Metrohm A.G.) was used as reference electrode and a Pt-wire electrode as counter-electrode. All the potential values were calculated versus Ag/AgCl, using the reference value + 0.21 V versus SHE at 20 °C [56].

To evaluate the ORR catalytic activity of our nanomaterials, we used the {Nafion:catalyst/carbon} glassy carbon electrode, mounted on the rotating-disk electrode (RDE) set-up. To evaluate the ORR kinetics, different rotation speeds were tested from 500 up to 3000 rpm of the RDE. The ORR measurements in acid conditions, were done in a O_2_-saturated cell containing 50 mL of triple distilled water, 0.1 M H_2_SO_4_ (pH = 1.3). At each rotation speed, ORR currents were measured versus the applied bias DC-potential, that was scanned linearly in the range E_bias_ = −0.2 V to +1 V, vs. Ag/AgCl, at a scan-speed of 10 mV/s. The ORR measurements in alkaline conditions were made in a O_2_-saturated cell, in 50 mL of triple distilled water, containing 0.1 M NaOH (pH = 13.4). At each rotation speed, ORR currents were measured versus the applied bias DC-potential that was varied linearly in the range of E_bias_ = −0.4 V to +0.4 V, versus Ag/AgCl, at a scan-speed of 10 mV/s. ORR polarization curves were recorded as Y = current density (J) vs. X = applied potential, were J was normalized was per geometric surface of the electrode, i.e., typically 0.071 cm^2^. According to the standard ORR procedures [31] the total mass (catalyst and carbon-support) per surface area was adjusted at 0.41 mg per cm^2^, allowing uninhibited conductivity between the glassy carbon and the active-phases, as well as proper signal-to noise [55]. Thus, in all our experiments discussed herein, the amount of the metal catalyst was 3 μg, accounting for 10% of the total {Nafion:catalyst/carbon} mass amount. Each electrochemical run was repeated for 50 repetitive cycle-scans.

Data analysis: The half-wave potential E_1/2_ is defined as the potential at which the measured current equals half the limiting current. Typically, E_1/2_ is used in ORR studies as a measure of the electrocatalytic activity, i.e., more positive E_1/2_ value, for the same rotation speed of the RDE, indicates a higher ORR activity [8,55,57]. In a given ORR data–set, recording *J* at various electrode rotation speeds (ω) provides data that, when analyzed according to Koutechy–Levich [54,55], provide information on the underlying electron-transfer characteristics of the ORR process [55]. The Koutechy–Levich equations are

(4) $$\frac{1}{J_{lim}}=\frac{1}{J_{k}}+\frac{1}{J_{l}}=\frac{1}{J_{k}}+\frac{1}{{B\omega }^{1/2}}$$

(5) B=0.62 n F D23 ν−16 Cb

(6) $$J_{k}={\mathrm{nFkC}}_{b}$$

In Equation (4) *J*_lim_ is the measured (limiting) current destiny in (mA/cm^2^), *J_k_* and *J_L_* are the kinetic- and diffusion- limiting current densities [43], and ω the rotation frequency of the RDE (in rpm). The kinetic current, *J_k_* is defined as the current that would be observed in the absence of mass-transport limitations, was calculated using Equation (4) from a plot of *J*_lim_ vs. (1/ω^1/2^). F is Faraday’s constant (96485 C mol^−1^), D is the oxygen diffusion coefficient (1.9 × 10^−5^ cm^2^ s^−1^) [43], *v* is the kinematic viscosity of the electrolyte that is (0.01 cm^2^ s^−1^) for 0.1 M NaOH and (0.03 cm^2^ s^−1^) for 0.1 M H_2_SO_4_ [43,58], C_b_ is the bulk concentration of oxygen (1.2 × 10^−6^ mol cm^−3^) [43], and *k* is the electron-transfer rate constant [43]. The integer n is the number of electrons transferred per reduced O_2_ molecule, that can be estimated using Equation (6) [43]. Notice that, it is the value of n, i.e., 2 or 4, that discriminates a two- or four-electron ORR process, as described in Equation (1).

## 3. Results

### 3.1. Characterization of the FSP-Made Cobalt-Oxide Nanocatalysts

Figure 1A shows XRD patterns for the #Co1, #Co2, and #Co3 materials. For #Co1, #Co2 the characteristic diffraction peaks at angles 19.1°, 31.3°, 36.8°, 38.8°, 44.6°, 55.8°, 59.5°, and 65.3° can be perfectly indexed to the (111), (220), (311), (222), (400), (422), (511), and (440) planes of Co_3_O_4_ (JCPDS card no. 75–2480) respectively [59]. For #Co3, additional characteristic peaks at 36.7°, 42.64°, 61.71°, 74.03°, and 77.98° can be indexed to the (111), (200), (220), (311) and (222) planes of CoO (JCPDS card no. 14–0133) respectively [60], indicating the phase composition of #Co3 was Co_3_O_4_/CoO. The CoO-phase was formed by Co^2+^ atoms, i.e., reduced Co^3+^, formed in-situ in FSP under our specific combustion conditions. Specifically, we have adjusted the fuel-to-oxygen equivalence ratio at φ = 0.9, which corresponds to an oxygen-lean combustion process, promoting the stabilization of reduced Co^2+^ and thus the CoO crystal formation, see Table 1. In the case of #Co1 material, the fuel-to-oxygen equivalence ratio was adjusted to φ = 0.7 which is oxygen-rich, thus no CoO-phase formation was observed, only Co_3_O_4_ phase was formed. In this way, adjustment of the combustion process parameters in FSP allowed a fine control of the Co_3_O_4_ & CoO nanophases. To examine the effect of a mild post-FSP calcination, i.e., that typically improves crystallinity in nanoparticles, a post-FSP calcination of #Co1 at T = 500 °C for 180 min was applied. As expected, calcination increased the primary particle size, i.e., *d*_XRD_ was increased from 10 nm in #Co1, to 27 nm in #Co2, with a concomitant decrease of the SSA from 100 m^2^/gr to 30 m^2^/gr, see Table 1.

Representative TEM images, depicted in Figure 1B–D, show high-quality crystal panes formation in the FSP-made materials #Co1 and #Co3 as well as in #Co2. Macroscopically, the nanoparticles were associated in fractal-like morphologies, typical for FSP-made metal-oxide particles [46]. Figure 1B–D show well-resolved lattice-fringes with d-spacing 0.28 nm, assigned to the (220) Miller-planes of Co_3_O_4_ phase in #Co1, #Co2 and #Co3 respectively [61]. In #Co3, the lattice-fringes with d-spacing 0.23 nm, in Figure 1D, are attributed to CoO crystal [62]. The particle size distribution, Figure 1E–G, show that #Co1 consisted of particles size in a range of 7–16 nm, with an average diameter *d_TEM_* = 10.5 nm, #Co2 had a wider range of particles size 20–60 nm with an average *d_TEM_* = 35.5 nm and #Co3 which had a wider range of particles size 29–60 nm with an average *d_TEM_* = 45 nm. Interestingly, in #Co2, i.e., calcined #Co1, the d*_XRD_* diameter, is smaller than the BET- or TEM-derived particle size, per Table 1. This is a nice demonstration that d*_XRD_* diameter is determined from the size of the primary crystalline size, while particle-sintering and agglomeration phenomena influence the BET and TEM derived particle size. An analogous observation holds true for the #Co3 material.

**Figure 2:**
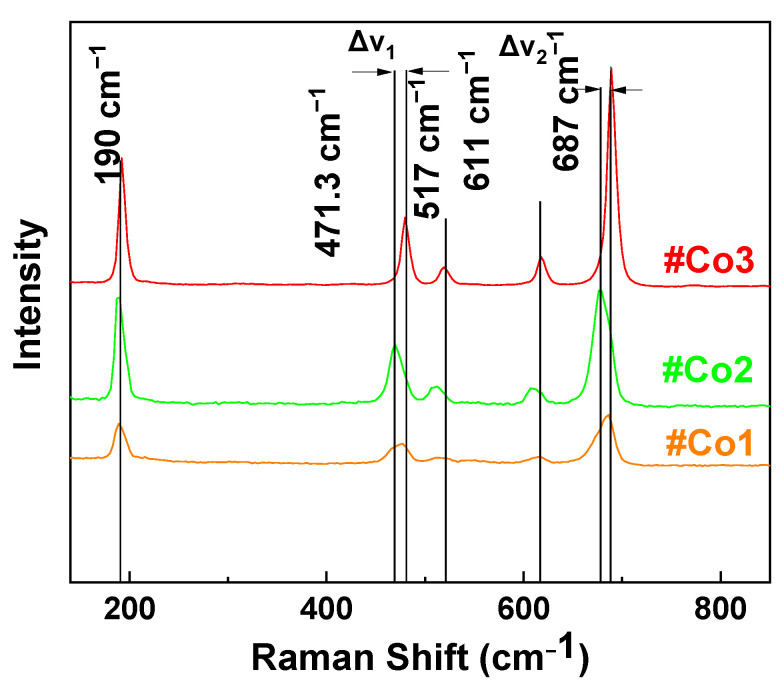
Raman spectra of the FSP-made Cobalt Oxide nanocatalysts.

*Raman Spectroscopy*: Figure 2 illustrates Raman spectra for the FSP-made Co-oxide nanomaterials. In all Raman spectra the characteristic vibration peaks of Co_3_O_4_ are prominent [60]. Specifically, the peaks at 189.5 cm^−1^, 471.3 cm^−1^, 516 cm^−1^, 610 cm^−1^, and 680 cm^−1^ in the spectra of #Co1 and #Co2 are assigned to the asymmetric- and bending-vibrations of Co_3_O_4_ spinel [63]. Upon calcination of #Co1, the improved crystallinity of #Co2 is manifested as sharped Raman peaks, plus some shifts on peaks at 480 cm^−1^ and 680 cm^−1^ band, i.e., compare #Co1 vs. #Co2.

Material #Co3 displayed distinct Raman peaks at 471.3 cm^−1^, 517 cm^−1^, 611 cm^−1^ and 687 cm^−1^, in accordance with a mixed-phase CoO/Co_3_O_4_ [60]. Notice that, the Raman peaks due to CoO are dominating the Raman spectrum of #Co3, despite the lower CoO percentage, i.e., estimated ratio CoO/Co_3_O_4_ is 34/66 according to XRD. Since the Raman scattering modes of CoO and Co_3_O_4_ are not expected to have fundamentally different intensity [60] we consider that the enhanced intensity of CoO in the Raman data in Figure 2, provides an indication of a core-shell configuration where CoO is the shell and Co_3_O_4_ is the core. Notice that the TEM images in Figure 1, despite their high resolution, do not allow a confirmative detection of core-shell structures, due to the very similar spacing of the Miller planes of CoO and Co_3_O_4_.

**Figure 3:**
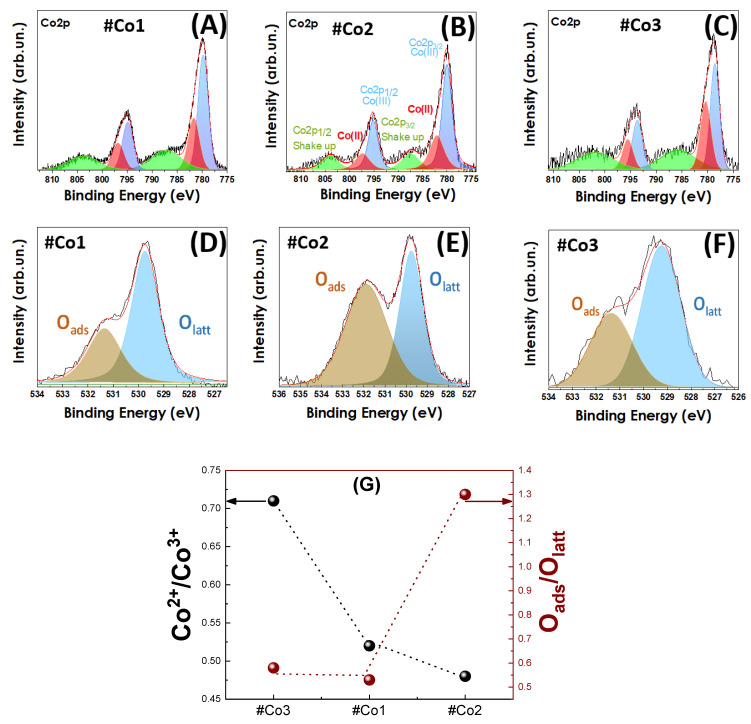
XPS spectra of (**A**,**D**) #Co1, (**Β**,**E**) post-FSP treated #Co2, (**C**,**F**) #Co3 XPS for core level of Co2 p_1/2_ and Co2 p_3/2_ (**A**–**C**) and (**D**–**F**) for the core level of O_1 s_. (**G**) the Co^2+^/Co^3+^ ratio, left Y-axis, and O_ads_/O_lattice_ ratio, right Y-axis, for the Co-oxide nanomaterials.

*X-Ray Photoelectron Spectroscopy:* XPS spectra, presented in Figure 3A–C, were recorded to probe the cobalt oxidation states and the associated oxygen vacancies. In accordance with literature XPS data [64,65], the Co2 p_3/2_ and Co2 p_1/2_ peaks are detected in Figure 3A–C, with the expected spin-orbit splitting ΔE = 15 eV. The peaks at 779.7 and 781.6 eV are assigned to Co^3+^ and Co^2+^ species respectively [64], as well as the peaks at 794.8 and 796.8 eV [64]. In the case of #Co3 material, the Co^2+^/Co^3+^ ratio was increased (see Table 1), indicating the presence of surface-related defects and/or tetrahedrally-coordinated Co^2+^ species. In order to clarify the origin of Co^2+^ presence, the XPS spectra for O1 s have been examined as well, see Figure 3D–F.

The dominant O-XPS peaks at 529.7 (blue) and 531.3 eV (brown) in Figure 3D–F, correspond to lattice-oxygen and adsorbed-oxygen species respectively [64,65]. As expected, the increased population of surface vacancies in #Co3 material promotes the Co^2+^ states, i.e., compare with #Co1 data in Figure 3D–F. In the case of post-FSP calcined #Co2 material, the increased percentage of adsorbed oxygen is correlated to the expected Co^3+^ formation during annealing. Figure 3G shows a correlation plot for the Co^2+^/Co^3+^ ratio, and O_ads_/O_lattice_ ratio, derived from the XPS data. Considering that the formal Co^2+^/Co^3+^ ratio for Co_3_O_4_ should be 1/2 = 0.5, i.e., each unit cell contains one Co^2+^ atom and two Co^3+^ atoms, the data in Figure 3G indicate a Co^2+^/Co^3+^ ratio of 0.52 for #Co1 and 0.48 for #Co2, i.e., both are close to 0.5 that is in agreement with the pure Co_3_O_4_. In an analogous-manner, material #Co3 with 34% CoO that is nominally consisting of 100% Co^2+^ centers, should have a formal Co^2+^/Co^3+^ ratio of 0.66 × (0.5) + 0.34 = 0.67. The data in Figure 3G verify this, i.e., #Co3 had a Co^2+^/Co^3+^ ratio 71%. Taking into account uncertainties in the phase-composition, i.e., by 5%, we consider that the Co^2+^/Co^3+^ ratio determined by XPS in in agreement with the phase-composition Co_3_O_4_/CoO~2/1 from XRD. Thus FSP-technology allows a precise control of phase-composition as well as Co^2+^/Co^3+^ ratio in Co_3_O_4_/CoO nanomaterials.

### 3.2. ORR Catalytic Results

#### 3.2.1. Oxygen Reduction Reaction

**Figure 4:**
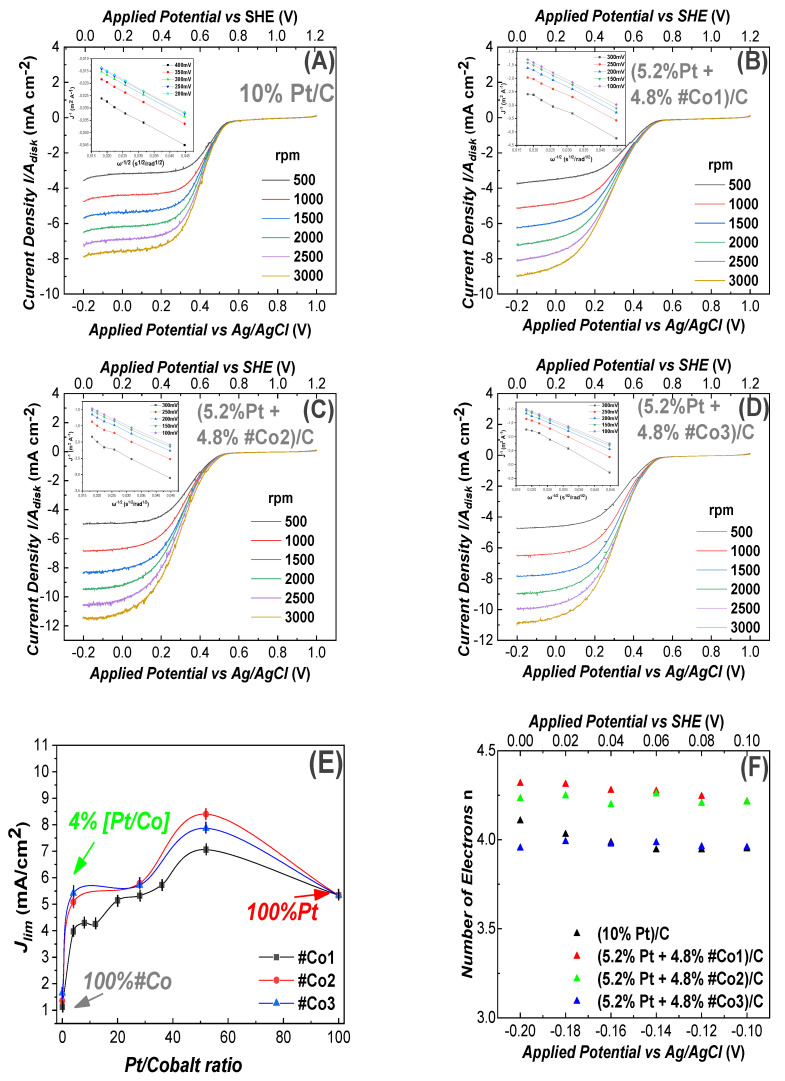
Linear Voltammograms for ORR, recorded using a Glassy-Carbon rotating electrode, loaded with nanocatalysts, in O_2_ saturated 0.1 M H_2_SO_4_ solution (pH = 1.3). The potential scan rate was 10 mV/s, (**A**) electrode containing 10% Pt/C (reference). In (**B**–**D**) all electrodes contained (5.2% Pt 4.8% FSP-Cobalt oxide/C). (**B**) #Co1-based electrode, (**C**) #Co2-based electrode, (**D**) #Co3-based electrode, (**E**) Limiting current density versus Pt/Cobalt ratio on the working electrodes, (**F**) Electron transfer number (n), estimated using Equation (6) for the data at 1500 rpm. *Insets in A, B, C, D*: Elovich plots J_lim_ vs. [rotation frequency]^−1/2^.

(1) ORR at Acidic pH: In Figure 4B–D, we present ORR data at acid pH for three working electrodes constructed using the Co-based materials. ORR data for the reference Pt-based electrode, are presented in Figure 4A. In all cases the total-metal loading of each electrode was 10%. In Figure 4B–D the electrode contained {4.8%Co-oxide+5.2% Pt}, i.e., almost half of the Pt mass vs. the reference Pt-based electrode. According to Figure 4B–D all our Co-based electrodes showed the same positive ORR half-wave potential (0.5 V vs. Ag/AgCl) in O_2_ saturated, 0.1 M H_2_SO_4_ (pH = 1.3) aqueous solution. The half-wave potential 0.5 V is in accordance with literature data for Cobalt-based electrodes [31]. The highest current density (J_lim_) was achieved by the #Co2-based electrode in Figure 4C, at a potential E = −200 mV, J_lim_ = 8.4 mA cm^−2^ at 1500 rad/s. This current density was higher than the J_lim_ = 5.57 mA cm^−2^ achieved by the reference Pt-electrode, at 1500 rad/s, see Figure 4A. Thus, incorporation of FSP-made Co_3_O_4_ nanoparticles into the ORR electrode had a significant positive effect in ORR. This trend was also observed at higher rotation-speeds. Herein, for the sake of the discussion, we exemplify/compare the ORR performance values at moderate rotation speeds, i.e., 1500 rpm. The #Co3-based electrode, Figure 4D, also achieved a high ORR activity at E = −200 mV with a current density of J_lim_ = 7.82 mA cm^−2^ at 1500 rad/s. Finally, the #Co1-based electrode, Figure 4B, achieved a current density of J_lim_ = 6.1 mA cm^−2^ at 1500 rad/s. Comparison of #Co2 with #Co1 shows that the post-FSP calcination process had a beneficial effect on the ORR performance. In all cases, in Figure 4A–D, increased rotation speeds exerted a strong beneficial influence on current densities, a well-known phenomenon for rotating-disk-electrodes [66] as predicted by Equations (4)–(6).

The linear Elovich plots, derived using Equation (6), see inset in each frame in Figure 4, allow an estimation of the number of transferred electrons. In all cases in Figure 4, the number of transferred electrons was consistently n~4, see Table 2. This indicates that our electrodes perform ORR via the, mostly desired, direct 4-electron process. The sigmoidal line-shape [61] of the curves in Figure 4, shows that the ORR process was diffusion-controlled for E < −0.1 V, mixed-diffusion kinetic-controlled in the potential region −0.1 to 0.1 V and kinetic-controlled in the potential region 0.53 to 0.60 V [61].

In Figure 4E, we compare the J_lim_ values for electrodes with different concentrations of Co-particles at 1500 rpm. We see that the highest J_lim_ current density was achieved when we used av ~ 50:50 Pt:Co-oxide ratio. Noticeably, our electrodes with minimal Pt of 0.4%, i.e., 9.6% #Co1 were also efficient, with an efficiency comparable to 10% Pt. The data in Figure 4E show that for a Pt-content of 5.2% (4.8% Co-oxide) the current density achieved was improved by ~30% vs the 10%Pt. Noticeably, at very low 0.4% Pt content, i.e., 9.6%Co-Oxide, all materials showed efficient ORR, with the best material #Co3 achieving J_lim_ = 5.71 mA/cm^2^. This is among the lowest Pt-contents reported so far in literature for metal-oxide nanoparticles, with common carbon-black/Vulcan as support. More specifically, to our knowledge, the best so far reported Metal-oxide with noble metal/ supported on Vulcan was an electrode with 75% Pt, i.e., (7.5 wt% Pd and 2.5 wt% Co)/C, which achieved a current density of J_lim_ = 1.8 mA/cm^2^ at acidic pH [31]. All the present Co-based electrodes were tested for up to 50 ORR cycles in acid pH and showed practically no-change in their performance profiles.

**Figure 5:**
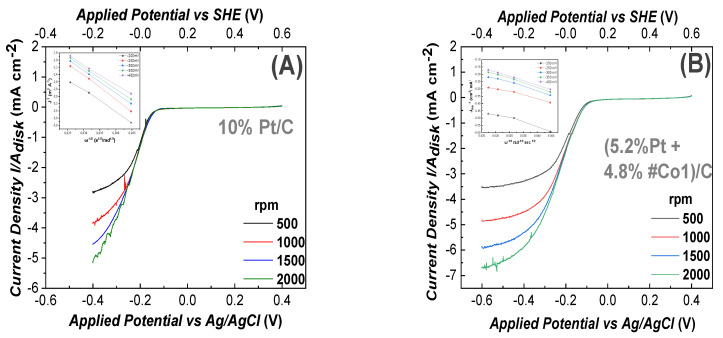

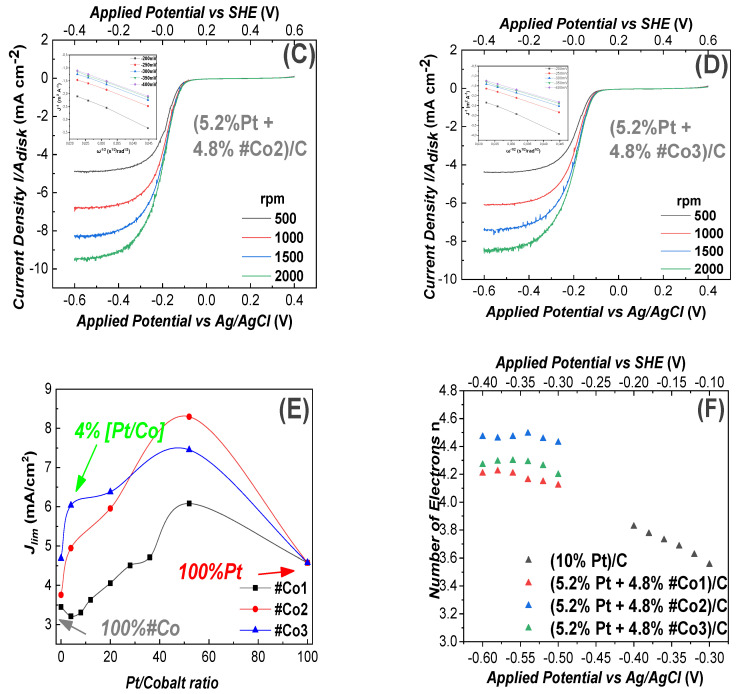
Linear Voltammograms for ORR, recorded using a Glassy-Carbon rotating electrode, loaded with nanocatalysts, in O_2_ saturated 0.1 M NaOH solution (pH = 13.4). The potential scan rate was 10 mV/s, (**A**) electrode containing 10% Pt/C (reference). In B, C, D all electrodes contained (5.2% Pt 4.8% FSP-Cobalt oxide/C). (**B**) #Co1-based electrode, (**C**) #Co2-based electrode, (**D**) #Co3-based electrode, (**E**) Limiting current density versus Pt/Cobalt ratio on the working electrodes, (**F**) Electron transfer number (n), estimated using Equation (7) for the data at 1500 rpm. *Insets in A, B, C, D*: Elovich plots where J_lim_ vs. [rotation frequency]^−1/2^.

(2) ORR at Alkaline pH: In Figure 5, we present ORR data at alkaline-pH 13.4. ORR data for the 100% Pt-based electrode as reference, are presented in Figure 5A. In all cases, the total-metal loading of each electrode was 10%. In Figure 5B–D the electrode contained 4.8%Co-oxide + 5.2% Pt, i.e., almost half of the Pt mass vs. the reference Pt-based electrode. According to Figure 5B–D all our Co-based electrodes showed the same negative ORR half-wave potential (−0.13 V vs. Ag/AgCl)) This is in accordance with literature data for Cobalt-based electrodes [67] in alkaline, O_2_ saturated, aqueous medium with 0.1 M NaOH (pH = 13.4). The highest current density J_lim_ = 8.31 mA cm^−2^ at 1500 rad/s was achieved by the #Co2-based electrode in Figure 5C, at a potential E = −600 mV. This current density is higher than the J_lim_ = 5.32 mA cm^−2^ at E = −400 mV, achieved by the reference Pt-electrode, at 1500 rad/s, see Figure 5A. This trend is also observed at higher rotation-speeds. The #Co3-based electrode, Figure 5D, achieved high activity at E = −600 mV with a limiting current density of J_lim_ = 7.38 mA cm^−2^ at 1500 rad/s. Finally, the #Co1-based electrode, Figure 5B, achieved a limiting current density of J_lim_ = 4.46 mA cm^−2^ at 1500 rad/s. Using the linear Elovich plots, see inset in each frame in Figure 5, using Equation (6), the number of transferred electrons was n~4, see Table 2.

As in the case of acid-medium, the sigmoidal shape [61] of the curves in Figure 5, means that, in alkaline medium, the ORR process was diffusion controlled for E<−0.55 V, mixed diffusion kinetic controlled in the potential region from −0.05 to 0 V and kinetic-controlled in the potential region from −0.55 to −0.40 V [61].

In Figure 5E, we compare the J_lim_ values for electrodes with Pt/Co ratio at 1500 rpm. We see that in alkaline, as well in acid medium, a Co/Pt ratio ~50:50 was optimal for the ORR. Noticeably, in alkaline medium, for a Pt-content of 5.2% and 4.8% #Co3, the maximum current density was improved by 24.7% vs. the 10%Pt. At very low Pt content, i.e., 0.4% Pt + 9.6%Co-Oxide, all materials showed efficient ORR, with the best material #Co3 achieving J_lim_ = 6.06 mA/cm^2^. All the present Co-based electrodes were tested for up to 50 ORR cycles in alkaline pH and showed practically no change in their performance profiles.

#### 3.2.2. Comparison with Literature

In Table 2, we present a comparison of ORR efficiency data, based on half wave potential and limiting current density J_lim_ (mA·cm^−2^) reported so far for pertinent cobalt-oxides on several supports.

According to Table 2, we can classify the ORR performances according to the pH, i.e., acid or alkaline. At alkaline pH, the best -so far reported- performance was achieved by a Co_3_O_4_ /rGO catalyst, that is Co_3_O_4_ supported on reduced Graphene-Oxide [62]. This material was reported to achieve a limiting current of 12.3 (mA·cm ^−2^) at 1600 rpm for a loading of 0.1 mg of catalyst per gr of electrode material. As analyzed by the authors [62] the reduced Graphene-Oxide played a determinative role in this ORR performance. Our best-performing electrode [5.2%Pt–4.8%#Co2/on Vulcan carbon black] achieved a limiting current of 8.3 (mA•cm^−2^) at 1500 rpm for a loading of 0.041 mg of catalyst per gr of electrode material. Taking into account the benefit of rGO used in [62] vs. Vulcan used in the present work, we consider that the FSP-made Co-oxide materials are highly promising for ORR. This is also evidenced by comparison of the present ORR data at alkaline pH vs. all other Co-based electrodes listed in Table 2. Concerning acid-pH, Table 2 shows that all our FSP-made catalysts supported on Vulcan, outperform all reported materials. Co_3_O_4_ has the normal-spinel structure Co^2+^Co_2_^3+^O_4_, in which the Co^3+^ ions occupy the octahedral sites, while the Co^2+^ ions occupy the tetrahedral sites, see inset structures in Figure 1 [76]. To further peer into the Oxygen Reduction Reaction, we carried out cyclic voltammograms (CV) experiments carried in O_2_ or N_2_ saturated cells. The data, presented in Figures S1 and S2 of Supplementary Materials, for 5.2% + 4.8% #Co2 show a reduction peak at 136 mV, and for and 5.2% + 4.8% #Co3 at 163 mV vs Ag/AgCl. These values are consistent with the redox activity of active sites associated with cations in the higher oxidation state (Co^3+^) at the cobalt oxide surface [69]. These Co^3+^ ions would act as donor-acceptor sites, i.e., electron acceptor role due to Co_3_O_4_ by capturing electrons, and electron-donor properties to O_2_ during oxygen reduction reaction [69]. We consider that the details of the Co-oxide nanofacets might also play a role in the ORR process. Theoretical calculation suggests that the three low Miller-index planes ({100}, {110} and {111}) of Co_3_O_4_ particles differ in the electronic structure, geometric bonding and chemical reactivity [77]. ORR is a surface reaction, i.e., O_2_ molecules can be preferably absorbed on active surface Co^2+^ (3d^5^4s^2^) sites, more than on surface Co^3+^ (3d^5^4s^1^) cations [71]. Our XPS analysis, revealed that in the present FSP Co_3_O_4_ -nanocatalysts O_2_ molecules are surface-absorbed via the Pauling mode (Co…(O_ads_ = O)) [78]. Taking into account our XPS and CV data, we consider that the interfacial active Co^2+^ sites are able to transfer electrons on the absorbed O_2_ molecules, i.e., to weaken the O-O bond and to assist breaking of O-O, while Co^2+^ is oxidized to Co^3+^ [43]. In this way, we consider that the presence Co^2+^ sites in the FSP-made materials, is of key importance for the observed ORR efficiency.

## 4. Conclusions

In the present work we have synthesized Co_3_O_4_ and CoO/Co_3_O_4_ nanostructures using flame spray pyrolysis (FSP). The FSP-made Co-Oxides were evaluated as electrocatalysts for ORR in acidic and alkaline medium. We have studied in detail the effect of Co-oxide/Pt ratio on ORR efficiency. We show that the FSP-made Co-oxides can perform ORR at very low-Pt loading, 0.4%. In acid medium an electrode with (5.2% Pt + 4.8% Co_3_O_4_), displayed the highest ORR performance (J_max_ = 8.31 mA/cm^2^, E_1/2_ = 0.66 V). In alkaline medium, superior performance and stability have been achieved by an electrode with (0.4% Pt + 9.6% [CoO/Co_3_O_4_]) with ORR activity (J_max_ = 3.5 mA/cm^2^, E_1/2_ = 0.08 V). XPS data suggest that Co^2+^ are promoted on the FSP-made Co-oxides and this shows a positive correlation with ORR activity. Comparison with literature shows that FSP-made Co-oxides can be considered highly promising for ORR technologies. For example, we envisage that their use with more efficient carbons supports, i.e., such as graphene or reduced graphene oxide, would further augment their efficiency. Technology-wise, the present work demonstrates that the developed FSP-protocols constitute a novel scalable process for the production of Co-oxides appropriate for oxygen reduction reaction electrodes.

## Figures and Tables

**Table 1:** Structural characterization data for the FSP-made Co_-_oxide nanomaterials.

*Material*	*d_BET_* *(nm ± 0.5)*	*d_XRD_* *(nm ± 0.5)*	*d_TEM_* *(nm ± 2)*	*SSA* *(m^2^ g^−1^ ± 5)*	*wt%Co_3_O_4_* (±5%)	*wt%CoO* (±5%)	Co^2+^/Co^3+^ *	O_ads_/O_latt_ *
Pt/C	3.5	4	-	100	-	-	-	-
Co#1	10	10	11	100	100	0	0.52	0.53
Co#2	32	27	37	30	100	0	0.48	1.3
Co#3	53	11(Co_3_O_4_) 9 (CoO)	44	18	66	34	0.71	0.58

* derived from XPS measurements.

**Table 2:** Comparison of ORR performance of the present FSP-Nanocatalysts vs. literature data for Co-based catalysts.

*Catalysts on GCE* *(Glassy Carbon* *Electrodes)*	*Catalyst* *Particle Size* *(nm)*	*Medium*	*Catalyst Loading* *(mg·cm^−2^)*	*e^−^* *Transferred*	*Half-Wave Potential vs SHE (V, rpm)*	*Catalytic Activity* *(mA·cm^−2^)*	*Mass Activity* *(A/mg_Pt_)*	*Ref.*
Co_3_O_4_ /rGO	12–25	Alkaline (0.1 M KOH)	0.120	3.9	0.79, 1600	12.3	-	[68]
Co_3_O_4_	31	Acid(BR buffer pH = 1.8)	0.643	2	0.71, 1250	0.6	-	[67]
Co_3_O_4_ −11	12	Alkaline (0.1 M KOH)	0.400	4	−0.052, 2400	3.5	-	[69]
CoO_x_ NPs/BNG	20–30	Alkaline (0.1 M KOH)	0.850	4	0.80, 1600	5.7	-	[70]
Co_3_O_4_/CNTs	24–28	Alkaline (0.1 M KOH)	0.150	4	0.80, 1600	5.2	-	[71]
Co-S/G−3 (nanosheets)	26–27	Alkaline (0.1 M KOH)	0.640	4	0.75, 1600	6.6	-	[72]
Co_2_O_3_@MF-C	44	Alkaline (0.1 M KOH)	0.051	4	0.85, 1600	5	-	[45]
Co-CoO@NC	15	Alkaline (0.1 M KOH)	0.100	4	0.80, 1600	4.8	-	[73]
Pt-Co Concave NCs/C	90	Acid (0.1 M HClO_4_)	0.153	4	0.90, 1600	6	0.26	[74]
Pt-CoO	16	Acid (0.1 M HClO_4_)	0.101	4	0.95, 1600	6.1	8.37	[75]
5.2% Pt–4.8%#Co1/Vulcan	10	Acid (0.1 M H_2_SO_4_)	0.041	4	0.66, 1500	6.3	0.029	this work
5.2%Pt–4.8% #Co1/ Vulcan	10	Alkaline (0.1 M NaOH)	0.041	4	0.08, 1500	5.9	0.028	this work
5.2%Pt–4.8%#Co2/Vulcan	27	Acid (0.1 M H_2_SO_4_)	0.041	4	0.66, 1500	8.3	0.039	this work
5.2%Pt–4.8% #Co2/ Vulcan	27	Alkaline (0.1 M NaOH)	0.041	4	0.08, 1500	8.3	0.039	this work
5.2%Pt–4.8%#Co3/ Vulcan	11	Acid (0.1 M H_2_SO_4_)	0.041	4	0.68, 1500	7.9	0.037	this work -
5.2%Pt–4.8%#Co3/ Vulcan	11	Alkaline (0.1 M NaOH)	0.041	4	0.08, 1500	7.4	0.035	this work

## Data Availability

Data is available upon the reasonable request from the corresponding author.

## Supplementary Materials

The following are available online at: https://www.mdpi.com/article/10.3390/nano11040925/s1, Figure S1: Cyclic Voltammetry curves of 5.2% Pt + 4.8% #Co2 at a scan rate of 20 mV s^−1^ in a O_2_ and N_2_ saturated cell in 0.1 M H_2_SO_4_ solution, Figure S2: Cyclic Voltammetry curves of 5.2% Pt + 4.8% #Co3 at a scan rate of 20 mV s^−1^ in a O_2_ and N_2_ saturated cell in 0.1 M H_2_SO_4_ solution.
