# Supplementary material for: Flame Spray Pyrolysis Co3O4/CoO as Highly-Efficient Nanocatalyst for Oxygen Reduction Reaction
Source: Nanomaterials (Basel). 2021 Apr 5;11(4):925. doi: 10.3390/nano11040925 (PMC8066371; doi:10.3390/nano11040925)
Supplement: Supplementary file 1 [file nanomaterials-11-00925-s001.pdf]

nanomaterials

*Article*

# Flame Spray Pyrolysis $Co_3O_4$/CoO as Highly-Efficient Nanocatalyst for Oxygen Reduction Reaction

**Loukas Belles [1], Constantinos Moularas [1], Szymon Smykała [2] and Yiannis Deligiannakis [1,*]**

1 Laboratory of Physics Chemistry of Materials & Environment, Department of Physics, University of Ioannina,45550 Ioannina, Greece; loukasbelles@gmail.com (L.B.); k.moularas@uoi.gr (C.M.).
2 Institute of Engineering Materials and Biomaterials, Silesian University of Technology, 18a Konarskiego St, 44-100 Gliwice, Poland; szymon.smykala@polsl.pl.
* Correspondence: ideligia@uoi.gr

Cyclic Voltammograms for #Co2 and #Co3 were recorder [scan rate of 20mV $s^{-1}$ ] in 0.1M $H_2SO_4$ solution saturated with $N_2$ or $O_2$. A well-defined cathodic peak at 0.14V vs Ag/AgCl can be seen for ORR in $O_2$-saturated 0.1M $H_2SO_4$ solution, which indicates that the catalysts have a good oxygen reduction electrocatalytic activity.

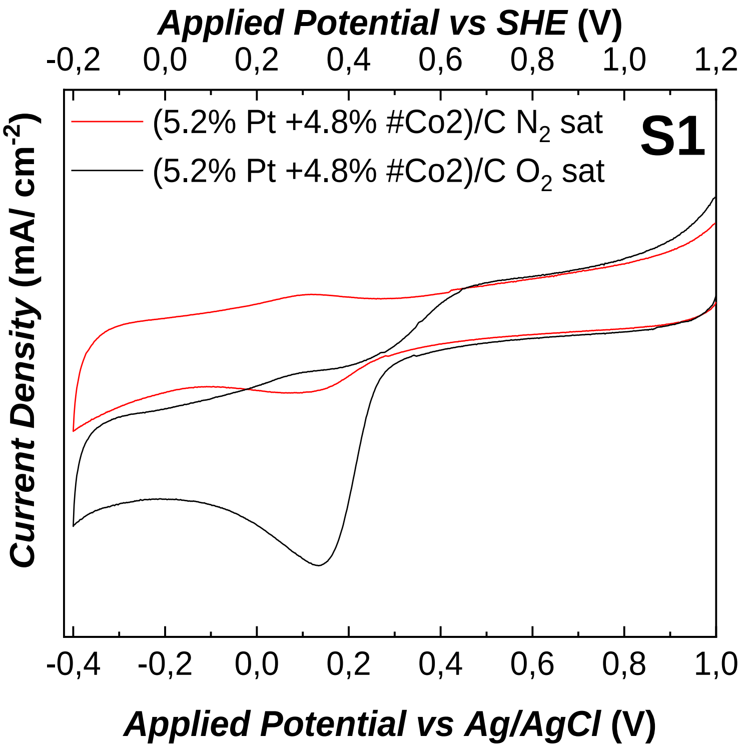


**Figure S1.** Cyclic Voltammetry curves of 5.2% Pt + 4.8% #Co2 at a scan rate of 20mV $s^{-1}$ in a $O_2$ and $N_2$ saturated cell in 0.1M $H_2SO_4$ solution.

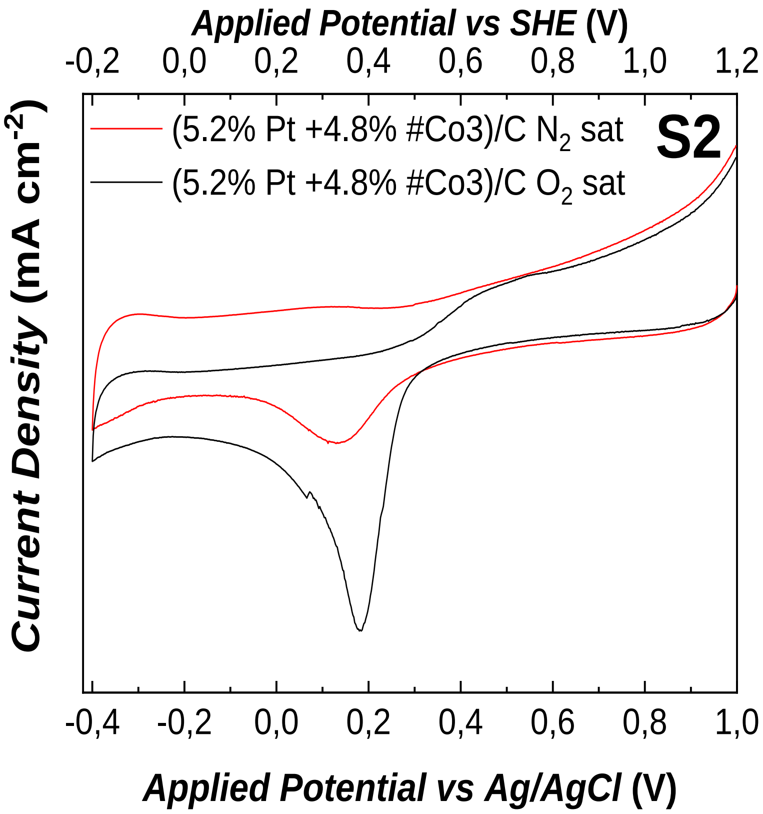


**Figure S2.** Cyclic Voltammetry curves of 5.2% Pt + 4.8% #Co3 at a scan rate of 20mV $s^{-1}$ in a $O_2$ and $N_2$ saturated cell in 0.1M $H_2SO_4$ solution.
